# Fatty Acid Metabolism-Related lncRNAs Are Potential Biomarkers for Predicting the Overall Survival of Patients With Colorectal Cancer

**DOI:** 10.3389/fonc.2021.704038

**Published:** 2021-08-11

**Authors:** Yurui Peng, Chenxin Xu, Jun Wen, Yuanchuan Zhang, Meng Wang, Xiaoxiao Liu, Kang Zhao, Zheng Wang, Yanjun Liu, Tongtong Zhang

**Affiliations:** ^1^ The Center of Gastrointestinal and Minimally Invasive Surgery, Department of General Surgery, The Third People’s Hospital of Chengdu, The Affiliated Hospital of Southwest Jiaotong University, Chengdu, China; ^2^ Department of Colorectal Surgery, National Cancer Center, National Clinical Research Center for Cancer, Cancer Hospital, Chinese Academy of Medical Sciences and Peking Union Medical College, Beijing, China; ^3^ Medical Research Center, The Affiliated Hospital of Southwest Jiaotong University, The Third People’s Hospital of Chengdu, Chengdu, China

**Keywords:** colorectal cancer, fatty acid metabolism, long noncoding RNA, ceRNA network, signature, nomogram

## Abstract

Abnormal metabolism, including abnormal fatty acid metabolism, is an emerging hallmark of cancer. The current study sought to investigate the potential prognostic value of fatty acid metabolism-related long noncoding RNAs (lncRNAs) in colorectal cancer (CRC). To this end, we obtained the gene expression data and clinical data of patients with CRC from The Cancer Genome Atlas (TCGA) database. Through gene set variation analysis (GSVA), we found that the fatty acid metabolism pathway was related to the clinical stage and prognosis of patients with CRC. After screening differentially expressed RNAs, we constructed a fatty acid metabolism-related competing endogenous RNA (ceRNA) network based on the miRTarBase, miRDB, TargetScan, and StarBase databases. Next, eight fatty acid metabolism-related lncRNAs included in the ceRNA network were identified to build a prognostic signature with Cox and least absolute shrinkage and selection operator (LASSO) regression analyses, and a nomogram was established based on the lncRNA signature and clinical variables. The signature and nomogram were further validated by Kaplan–Meier survival analysis, Cox regression analysis, calibration plots, receiver operating characteristic (ROC) curves, decision curve analysis (DCA). Besides, the TCGA internal and the quantitative real-time polymerase chain reaction (qRT-PCR) external cohorts were applied to successfully validate the robustness of the signature and nomogram. Finally, *in vitro* assays showed that knockdown of prognostic lncRNA TSPEAR-AS2 decreased the triglyceride (TG) content and the expressions of fatty acid synthase (FASN) and acetyl-CoA carboxylase 1 (ACC1) in CRC cells, which indicated the important role of lncRNA TSPEAR-AS2 in modulating fatty acid metabolism of CRC. The result of Oil Red O staining showed that the lipid content in lncRNA TSPEAR-AS2 high expression group was higher than that in lncRNA TSPEAR-AS2 low expression group. Our study may provide helpful information for fatty acid metabolism targeting therapies in CRC.

## Introduction

Worldwide, colorectal cancer (CRC) is the third most common cancer and the second leading cause of cancer-related death (1). It has been estimated that more than 1.8 million patients are diagnosed with CRC, and approximately 900,000 people die from CRC annually, primarily (2, 3). Despite rapid advances in detection and treatment, the mortality of CRC remains high due to the lack of biomarkers for early screening and prognosis prediction, meaning that many cases are not diagnosed until advanced clinical stages (3). Therefore, efficient prognostic biomarkers and a greater understanding of the molecular mechanisms of CRC are essential to improve the prognosis of patients with CRC.

Energy metabolism reprogramming, which can promote rapid cell growth and proliferation, is an emerging hallmark of cancer (4, 5). Cancer cells are known to be prone to the “Warburg effect”, which is enhanced glycolysis or aerobic glycolysis (6). In addition to the dysregulation of glucose metabolism, abnormal fatty acid metabolism has gained increasing attention in recent years as a characteristic of metabolic reprogramming in cancer (7–9), especially in CRC (10, 11). In oncogenesis, fatty acids are needed to store energy, synthesize membranes, and produce signaling molecules (12). It has shown that the decipherment of the fatty acid metabolism and molecular mechanism of CRC will lead to identifying novel therapeutic targets, thus developing effective treatment methods (13, 14). Wang et al. found that CPT1A-mediated fatty acid oxidation activation inhibits anoikis in CRC cells, which indicated that CPT1A is an attractive target to treat metastatic CRC (15). However, the regulatory mechanism of fatty acid metabolism pathway in CRC has not been deeply studied. Therefore, the identification of fatty acid metabolism-related genes might generate new avenues to explore with regard to the treatment of CRC.

Long noncoding RNAs (lncRNAs), defined as non-protein-coding RNA transcripts of over 200 nucleotides, are involved in gene regulation and various cellular processes (16, 17). Dysregulation of lncRNAs is widespread in cancer and has been shown to promote tumorigenesis and progression (18). Many researchers have explored the lncRNA expression profiling of CRC and found they can serve as biomarkers of CRC prognosis and diagnosis (19, 20). Notably, recent investigations have demonstrated that lncRNAs can affect the progression of cancer by altering the fatty acid metabolism (21–23). The competing endogenous RNA (ceRNA) hypothesis proposed by Salmena et al. is based on a large-scale regulatory network system representing the complex cross talk between coding and non-coding RNAs (24). According to the hypothesis, lncRNAs may regulate fatty acid metabolism-related mRNA expression by sponging miRNAs in CRC. Although a few studies have constructed ceRNA networks in CRC (19, 25, 26), the regulatory function of fatty acid metabolism-related ceRNA remains unclear.

In this study, we first obtained data from the KEGG pathway and The Cancer Genome Atlas (TCGA) database. The gene set variation analysis (GSVA) enrichment score of the fatty acid metabolism pathway in each CRC sample was calculated to inquiry the correlation between the fatty acid metabolism pathway and CRC. We next applied bioinformatics methods to construct a fatty acid metabolism-related ceRNA network. Subsequently, we identified eight fatty acid metabolism-related lncRNAs associated with survival included in the ceRNA network and constructed a prognostic signature. Then we combined the lncRNA signature with clinical variables to establish a nomogram to enhance the accuracy of survival prediction for CRC patients. The nomogram was validated using both TCGA internal and quantitative real-time polymerase chain reaction (qRT-PCR) external cohorts. Finally, we chose lncRNA TSPEAR-AS2 included in the signature to demonstrate its key role in the fatty acid metabolism of CRC.

## Materials and Methods

### Data Collection and Preprocessing

**Figure 1:**
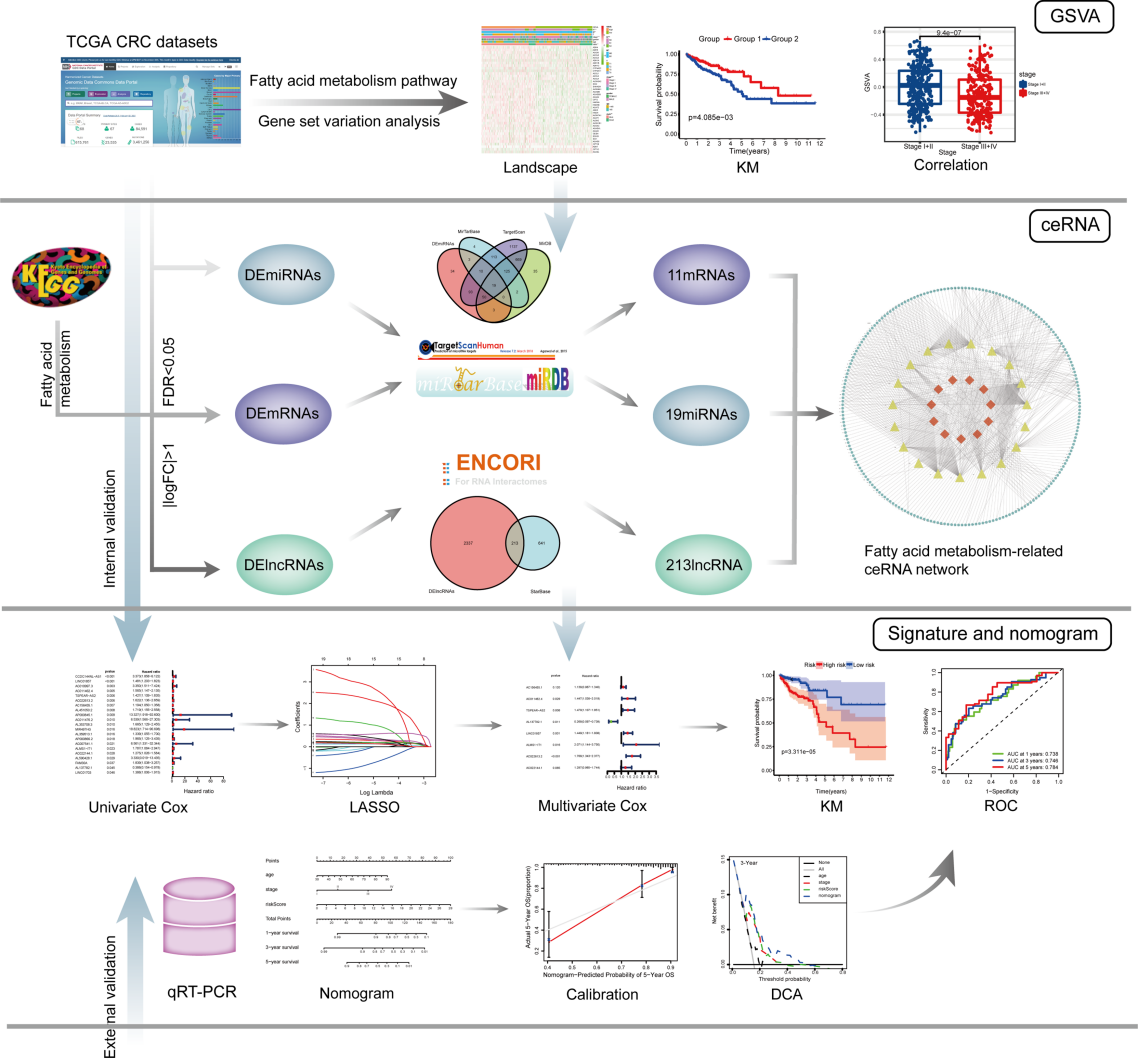
The entire analysis process of this study.

The entire analysis process of this study is shown in **Figure 1**.

The RNA sequencing (RNA-Seq) data, including mRNA and lncRNA expression data and miRNA expression data of CRC samples, were downloaded from “TCGA-COAD” and “TCGA-READ” projects of the TCGA database^1^. These data were in the format of HT-Seq raw read count and log_10_-transformed and normalized by the “limma (27)” R package in R (version 3.6.1). LncRNAs and mRNAs were annotated according to the Genome Reference Consortium Human Build 38 (GRCh38) from the GENCODE website^2^. Perl (version 5.28.1) was used for data processing. As a result, 14,142 lncRNA, 2158 miRNA, and 19,658 mRNA profiles were obtained from TCGA database. Clinical data of 548 CRC samples, including survival information, age, sex, tumor stage, and TMN stage, were also downloaded from “TCGA-COAD” and “TCGA-READ” projects of the TCGA database. We screened 506 samples according to the following inclusion criteria: (1) samples with complete overall survival (OS) and survival state; (2) samples with OS ≥ 30 days; and (3) samples with complete RNA-Seq data. The clinical information of 506 TCGA colorectal cancer samples is shown in **Table 1**. The list of fatty acid metabolism-related genes (refer to the genes included in the fatty acid metabolism pathway) was extracted from the map01212 gene set of the Kyoto Encyclopedia of Genes and Genomes (KEGG) gene set in the KEGG database^3^, and the expression levels of 42 genes were available in the expression profile from TCGA.

**Table 1:** The clinical information of 506 TCGA colorectal cancer samples.

Characteristics	Samples (n = 506)
Age (years)	
≤ 65	225
> 65	281
Sex	
Male	276
Female	230
Survival status	
Alive	415
Dead	91
Tumor stage	
I	90
II	188
III	140
IV	73
unknown	15
Tumor invasion depth	
T1	14
T2	91
T3	344
T4	56
Tis	1
Lymph node status	
N0	295
N1	124
N2	86
Nx	1
Metastasis status	
M0	379
M1	72
Mx	49
unknown	6

### Gene Set Variation Analysis and Clinical Correlation Analysis

The GSVA enrichment score of the fatty acid metabolism pathway in each CRC sample was calculated using the “GSVA (28)” R package. We classified the samples into two groups based on the median cutoff of the GSVA enrichment score. The expression of fatty acid metabolism-related genes was visualized in a heatmap using the “pheatmap” R package. The correlation between the group and clinical variables was analyzed by Chi-square test (using the R language). Kaplan–Meier survival analysis was applied to analyze the difference of OS between the two groups (using “survival” and “survminer” R packages). Additionally, the “ggplot2” R package was used to draw boxplots and analyze the differences in GSVA enrichment scores between different subgroups.

### Analysis of Differential Expression

Differentially expressed lncRNAs, miRNAs, and fatty acid metabolism related-mRNAs (referred to as DElncRNAs, DEmiRNAs, and DEmRNAs, respectively) were identified by the “limma” R package according to the following criteria: false discovery rate (FDR) < 0.05 and |logfold change (FC)| > 1. The volcano plots of differentially expressed RNAs were drawn using the “ggplot2” R package.

### Construction of the ceRNA Network

Fatty acid metabolism-related DEmRNAs targeted by DEmiRNAs were retrieved based on the miRTarBase^4^, miRDB^5^, and TargetScan^6^ databases, and only overlapping parts of the three databases served as candidate DEmiRNAs. The StarBase^7^ database was used to predict the interactions between DElncRNAs and candidate DEmiRNAs. Cytoscape (version 3.6.1) was applied to establish the ceRNA network for visualization.

### Construction and Validation of the lncRNA-Based Prognostic Signature

The 506 complete CRC samples were randomly classified into a training cohort (n =354) and a validation cohort (n =152) using the “caret” R package, with a ratio of 7:3. The clinical information of colorectal cancer samples in the TCGA training cohort and validation cohort is shown in **Table 2**. In the training cohort, univariate Cox regression analysis was applied to identify the OS-related lncRNAs (*P* < 0.05) in the ceRNA network, which were subsequently used for the LASSO regression analysis. Then the regression coefficients of the prognostic signature were determined by a multivariate Cox regression model (using “glment”, “survminer”, and “survival” R packages). The corresponding hazard ratios (HRs), 95% confidence intervals (CIs), and *P*-values were also calculated and presented in a forest plot. The risk score of each patient was calculated as follows:


$$Risk\;Score\left(RS\right)=\sum_{i}^{n}\left({Exp}_{i}*{Coef}_{\text{i}}\right)$$

where *Expi* represents the expression level of risk lncRNAs, *Coefi* represents the regression coefficient of risk lncRNAs, and *n* is the number of risk lncRNAs. Based on the median risk score, all samples were classified into high- and low-risk groups, which were subsequently used for Kaplan–Meier survival analysis. Time-dependent ROC curves and a risk heatmap were developed to assess the efficiency of the signature using “survivalROC” and “heatmap” R packages. Finally, the TCGA validation cohort and the qRT-PCR cohort were used to test the versatility and reliability of the signature internally and externally with the same analysis process, respectively.

**Table 2:** Clinical information of colorectal cancer samples in the TCGA training cohort and TCGA internal validation cohort.

Characteristics	Training cohort (n = 354)	Internal validation cohort (n = 152)
Age (years)		
≤ 65	157	68
> 65	197	84
Sex		
Male	187	89
Female	167	63
Survival status		
Alive	297	118
Dead	57	34
Tumor stage		
I	70	20
II	132	56
III	100	40
IV	43	30
unknown	9	6

### Construction and Validation of a Nomogram

Kaplan–Meier survival analysis was performed to identify the significant differences in OS between different subgroups to detect meaningful clinical variables. Meanwhile, the univariate and multivariate Cox regression analyses included risk score, age, sex, and tumor stage. We chose the variables with a *P* < 0.05 in the multivariate Cox analysis to build a novel nomogram. The calibration plots were drawn using the “rms” R package. Based on the nomogram, the total risk score was calculated and used to classify the training cohort into two groups based on the median cutoff, which were subsequently used for Kaplan–Meier survival analysis. ROC curves were drawn to compare the predictive power of the nomogram and clinical variables by the “survivalROC” R package. Besides, DCA, a method for assessing prediction models (29), was conducted to compare the clinical applicability of the nomogram with other clinical variables by calculating the net benefit using the “stdca” R package. Finally, the prognostic nomogram was internally verified using the TCGA validation cohort and externally verified using the qRT-PCR cohort with the same approach.

### Clinical Samples and Quantitative Real-Time PCR

We collected frozen and surgically resected tumor tissues from 110 patients with pathologically diagnosed CRC at the Cancer Hospital Chinese Academy of Medical Sciences. After removal, the surgical specimens were immediately frozen in liquid nitrogen and stored at −80°C.

Total RNA was reverse-transcribed into cDNA with transcriptor first strand cDNA synthesis kit (Roche, Penzberg, Germany) according to the manufacturer’s instructions. qRT-PCR analysis was performed using the FastStart Essential DNA Green Master mix (Roche, Penzberg, Germany) on a Roche LightCycler 480 (Roche, Penzberg, Germany) to measure the expression levels of lncRNAs. The expression levels of the identified lncRNAs were normalized to GAPDH and analyzed by the 2−ΔΔCt method. We verified the specificity of the designed primers by BLAST. A single peak of the melting curve implied a specific product. Each sample was performed in triplicate. The primers utilized in this study are shown in **Supplementary Table S1**. The clinical information and qRT-PCR results of colorectal cancer samples in qRT-PCR external validation cohort are shown in **Supplementary Table S2**.

### Cell Line and Culture

CRC cell lines, SW480 and SW620 were purchased from Cell Bank of Type Culture Collection, Chinese Academy of Sciences (Shanghai, China) and cultured in RPMI 1640 medium supplemented with 10% FBS at 37°C in 5% CO2.

### IncRNA Knockdown

Small interfering RNA (siRNA) of TSPEAR-AS2 were designed by GenePharma (Shanghai, China). Cells were transfected for 48 h using Lipofectamine 2000 (Invitrogen, CA, USA). Knockdown efficiency was evaluated by qRT-PCR. The sequence of siRNA was 5’-AGAAUGAGAAGAUCAGAACUG-3’.

### Western Blot Analysis

Proteins were extracted from cells using RIPA lysis buffer. Protein concentration was determined using the bicinchoninic acid protein assay kit (Sigma). The extracts were separated by 12% SDS-PAGE and transferred to PVDF membranes. After blocking for 1h, the membranes were incubated with the primary anti-fatty acid synthase (FASN) and acetyl-CoA carboxylase 1 (ACC1) antibodies (Cell Signaling Technology, Danvers, MA, USA) at 4°C overnight. Then the membranes were incubated with HRP-conjugated secondary antibody at room temperature for 2h. To visualize signals, ECL was conducted with GAPDH as an endogenous protein for normalization.

### Intracellular Triglyceride Assay

Intracellular triglyceride was determined using an E1013 triglyceride assay kit (Applygen Technologies, Beijing, China) at 550nm. After the cells were lysed and left standing for 10 minutes, the supernatant was taken and transferred to a 1.5 mL centrifuge tube. Then the liquid was heated at 70°C for 10 min and centrifuged at 2000rpm for 5 minutes. Finally, the supernatant was used for enzymatic determination.

### Chromogenic *In Situ* Hybridization (CISH)

To verify the expression of lncRNA TSPEAR-AS2 in CRC, digoxigenin (DIG)-labeled CISH was conducted on tumor tissues. The samples were fixed with 4% paraformaldehyde for 2-12 h. And we prepared paraffin sections for hybridizations. Then, we put the sections in boiling water for 15 min and cooled them at room temperature. The specimens were incubated with Proteinase K (Servicebio) at 37°C for 30 min and rinsed three times in PBS (Servicebio). We conducted prehybridization at 37°C for 1 h in hybridization buffer (Servicebio). Then, the samples were incubated overnight at 37°C with fresh hybridization buffers containing 8ng/ml probes instead of prehybridized buffers. The washed specimens were incubated at room temperature in blocking serum containing BSA for 30 min, followed by incubation at 37°C for 40 min with anti-DIG/AP antibody (Jackson). Probes labeled with DIG resulted in permanent diaminobenzidine (DAB) brown-colored, distinct, dot-shaped signals.

### Oil Red O Staining

The lipid deposits in tumor tissues with high and low expression of TSPEAR-AS2 were visualized by Oil Red O staining. Tumor tissue sections were stained with 30% Oil Red O in isopropanol for 60 min. The lipid deposition of the tissue sections was observed under light microscopy. Then the stained Oil Red O was dissolved using isopropanol for 5 min. The lipid concentrations were determined by the absorbance of the extract at 540 nm.

## Results

### Clinical Correlation of the Fatty Acid Metabolism Pathway in Patients With Colorectal Cancer

**Figure 2:**
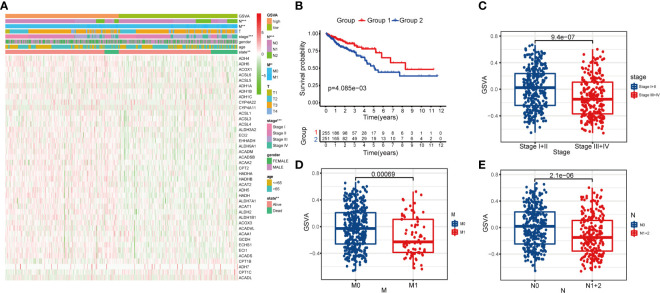
Clinical correlation of fatty acid metabolism pathway in patients with CRC. **(A)** The heatmap showing the associations between the GSVA enrichment score of fatty metabolism pathway and CRC-related clinical characteristics in the dataset from TCGA. **p < 0.01; ***p < 0.001. **(B)** Kaplan–Meier curves of OS in TCGA-CRC patients based on the GSVA enrichment score. **(C–E)** boxplots comparing the GSVA enrichment scores in different tumor stages, M stages, and N stages of TCGA-CRC patients.

After calculating the GSVA enrichment score of the fatty acid metabolism pathway of each CRC patient, we found that the score was closely related to the tumor stage (*P* < 0.001), metastasis status (M) (*P* < 0.01), and lymph node status (N) (*P* < 0.001) (**Figure 2A**). We classified the CRC samples into two groups by the median cutoff of the GSVA enrichment score. Surprisingly, the OS was significantly different between the two groups by Kaplan–Meier survival analysis (*P* = 4.085e−03) (**Figure 2B**). Notably, the boxplots show patients with different tumor stages, M, and LN status had significantly different GSVA enrichment scores (**Figures 2C–E**).

### Identification of Differentially Expressed Genes

**Figure 3:**
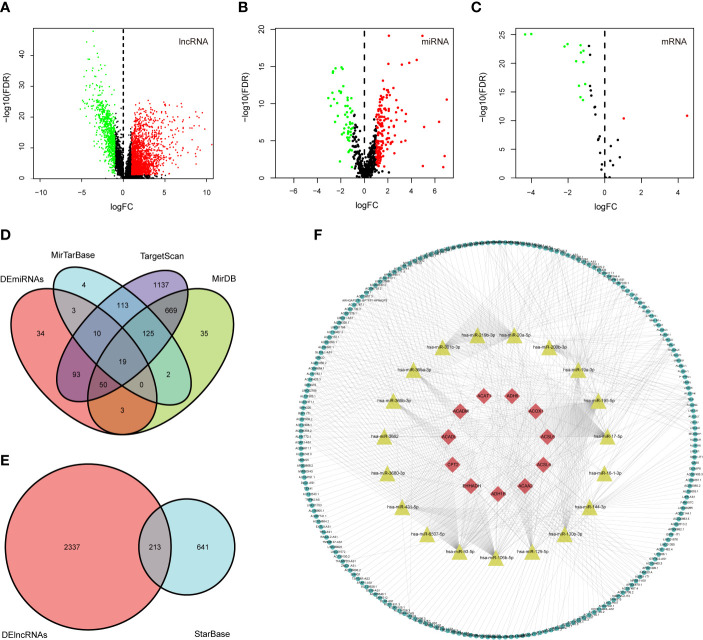
Construction of the fatty acid metabolism-related ceRNA network. **(A–C)** Volcano plots of differential expression of lncRNAs, miRNAs, and fatty acid metabolism related-DEmRNAs. Red indicates upregulated RNAs, and green indicates downregulated RNAs; **(D)** the intersection of predicted miRNAs and DEmiRNAs; **(E)** the intersection of predicted lncRNA and DElncRNAs; **(F)** Fatty acid metabolism-related ceRNA network. Red nodes represent the 11 intersected DEmRNAs. Yellow nodes represent the 19 intersected DEmiRNAs. Green nodes represent the 213 intersected DElncRNAs.

DElncRNAs and DEmRNAs were screened from 568 CRC samples and 44 adjacent normal samples; DEmiRNAs were screened from 446 CRC samples and 45 adjacent normal samples. Based on the cutoff values of |logFC | > 1 and FDR < 0.05, we identified 2550 DElncRNAs (739 downregulated and 1811 upregulated), 212 DEmiRNAs (53 downregulated and 159 upregulated), and 13 fatty acid metabolism-related DEmRNAs (13 downregulated and 2 upregulated). The volcano plots for differentially expressed RNAs between CRC samples and adjacent normal samples are shown in **Figures 3A–C**.

### Construction of the ceRNA Network

We mapped the 15 fatty acid metabolism-related DEmRNAs into the TargetScan, miRDB, and miRTarBase databases to predicted targeting miRNAs; 144 miRNAs that interacted with 11 of the 14 DEmRNAs in all 3 datasets were selected, and after taking the intersection with 212 DEmiRNAs, 19 out of predicted miRNAs were selected (**Figure 3D**). We then used the StarBase database to predict which of the lncRNAs and 19 miRNAs could interact with each other. As a result, 213 of the predicted lncRNAs were selected after taking the intersection with 2550 DEmiRNAs (**Figure 3E**). Finally, a ceRNA network including 213 DElncRNAs, 19 DEmiRNAs, and 11 DEmRNAs was eventually constructed using Cytoscape software (**Figure 3F**).

### Construction of the lncRNAs Prognostic Signature

**Figure 4:**
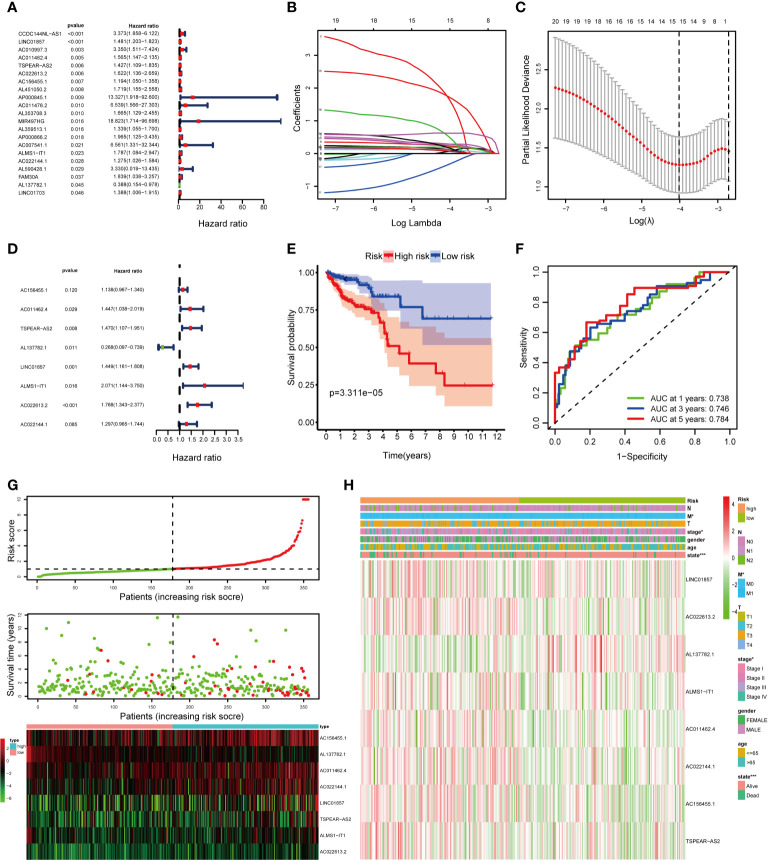
Construction of the fatty acid metabolism-related lncRNAs prognostic signature in the training cohort. **(A)** Univariate Cox regression analysis selected 21 fatty acid metabolism-related lncRNAs correlated with survival; **(B)** LASSO coefficient profiling of the 21 fatty acid metabolism-related lncRNAs; **(C)** A coefficient profile plot was generated against the log (lambda) sequence; **(D)** multivariate Cox regression analysis selected 8 fatty acid metabolism-related lncRNAs correlated with survival; **(E)** Kaplan–Meier curves of patients with CRC based on the prognostic signature; **(F)** ROC curves of 1-, 3-, and 5-year OS predicted by the prognostic signature; **(G)** The distribution of the risk score, OS, and lncRNA expression pattern; **(H)** The heatmap showing the associations between the risk score and CRC-related clinical variables. *p < 0.05; ***p < 0.001.

We classified the 506 complete CRC samples randomly into a training cohort (n =354) and a validation cohort (n =152), with a ratio of 7:3. To consider whether the 213 DElncRNAs included in the ceRNA network were closely associated with OS of CRC samples in the training cohort, univariate Cox regression analysis was performed to determine the significant DElncRNAs. Twenty-one DElncRNAs related to the OS (*P* < 0.05) were identified and applied to the following analysis (**Figure 4A**). Next, we conducted the LASSO regression model analysis, which identified 15 crucial DElncRNAs (**Figures 4B, C**). Finally, through multivariate Cox regression analysis, we screened eight lncRNAs to construct a prognostic signature for CRC (**Figure 4D**).

The risk score of each patient was calculated as follows: risk score = (0.12955 × expression level of AC156455.1) + (0.36982 × expression level of AC011462.4) + (0.38508 × expression level of TSPEAR-AS2) – (1.31514 × expression level of AL137782.1) + (0.37069 × expression level of LINC01857) + (0.72797 × expression level of ALMS1-IT1) + (3.32329 × expression level of AC022613.2) + (0.26023 × expression level of AC022144.1). After scoring each patient’s risk through the signature, we divided the CRC samples into high- and low-risk groups by the median cutoff (**Figure 4G**). We found that the OS of the high-risk group was significantly lower than that of the low-risk group (*P* = 3.311e−05) by Kaplan–Meier survival analysis (**Figure 4E**). Moreover, the AUCs were assessed for 1-year (AUC = 0.738), 3-year (AUC = 0.746), and 5-year (AUC = 0.784) survival (**Figure 4F**), and the results demonstrated promising predictive value for CRC patients. Besides, the clinical variables were then compared between the two groups. We found significant differences between the two groups in tumor stage (*P* < 0.05), M (*P* < 0.05), and state (*P* < 0.001) (**Figure 4H**).

### Construction of a Nomogram

**Figure 5:**
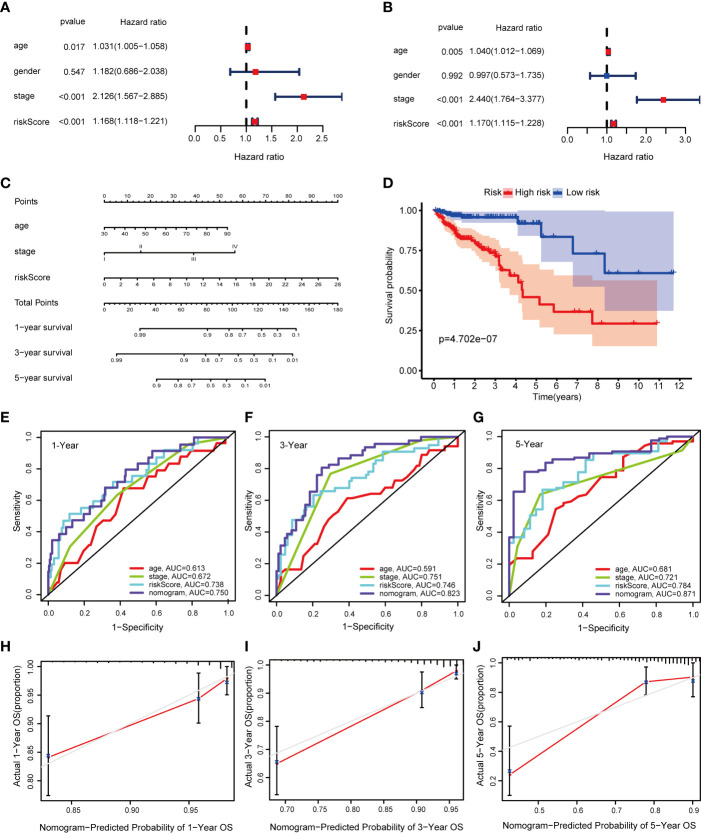
Construction of a nomogram in the training cohort. **(A, B)** Univariate and multivariate Cox regression analysis of OS-related variables; **(C)** The nomogram consists of age, tumor stage, and risk score; **(D)** Kaplan–Meier curves of patients with CRC based on the nomogram; **(E–G)** ROC curves of 1-, 3-, and 5-year OS predicted by the nomogram; **(H–J)** Calibration curves of 1-, 3-, and 5-year OS predicted by the nomogram.

To identify meaningful clinical variables, we evaluated the effects of age, sex, and tumor stage using Kaplan–Meier survival analysis. We found that age and tumor stage are of great significance to the prognosis of CRC (**Supplementary Figures 1A–C**). We screened the significant variables in the training cohort through univariate and multivariate Cox regression analyses (**Figures 5A, B** **)**. The results demonstrated that the prognostic signature is an independent clinical prognostic variable (*P* < 0.001) for patients with CRC. To facilitate the application of our signature in the clinical prognosis of patients with CRC, we chose the variables with a *P* < 0.05 in the multivariate Cox analysis, including age, tumor stage, and risk score, to build a nomogram to predict the OS of CRC patients (**Figure 5C**). Then the total risk score was calculated according to each prognostic variable in the nomogram. Based on the median cutoff, we classified the patients into high- and low-risk groups, and the OS was better in the low-risk group than in the high-risk group (*P* = 4.702e−07) (**Figure 5D**). Moreover, the AUCs were evaluated for 1-year (AUC = 0.750), 3-year (AUC = 0.823), and 5-year (AUC = 0.871) survival (**Figures 5E–G**). The ROC curves also indicated that the nomogram combining the signature and clinical variables had greater predictive accuracy than a single clinical variable. Finally, the calibration curve for OS was applied to evaluate the predictive value of the nomogram, where the gray line indicates the actual observation (**Figures 5H–J**). The results of the OS prediction indicated strong consistency between the nomogram prediction and the actual observation. Finally, the DCA was applied to compare the net benefit of the nomogram with clinical variables to patients under different threshold probabilities. The DCA curves of 1-, 3-, and 5-year OS for the nomogram and other clinical variables are shown in **Supplementary Figures 2A–C**. The results show that the nomogram has a better net benefit at different threshold probabilities, indicating that the nomogram has superior clinical practicability.

### Validation of the Signature and Nomogram in the Internal and External Validation Cohorts

**Figure 6:**
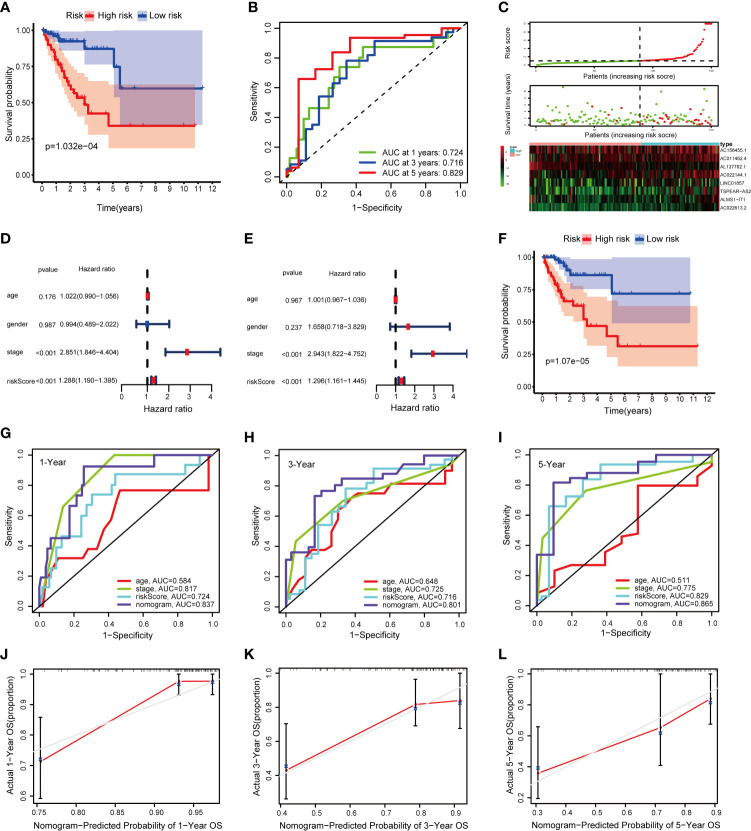
Validation of the signature and nomogram in the TCGA internal validation cohort. **(A)** Kaplan–Meier curves of patients with CRC based on the prognostic signature; **(B)** ROC curves of 1-, 3-, and 5-year OS predicted by the prognostic signature; **(C)** The distribution of the OS, risk score, and lncRNA expression pattern; **(D, E)** Univariate and multivariate Cox regression analysis of OS-related variables; **(F)** Kaplan–Meier curves of patients with CRC based on the nomogram; **(G–I)** ROC curves of 1-, 3-, and 5-year OS predicted by the nomogram; **(J–L)** Calibration curves of 1-, 3-, and 5-year OS predicted by the nomogram.

The TCGA internal validation was utilized to test the validity and robustness of the signature and nomogram by the same analysis process. We calculated the risk score based on the fatty acid metabolism-related lncRNAs prognostic signature for each patient in the internal validation cohort by the same formula and classified them into low- and high-risk groups by the median cutoff (**Figure 6C**). As expected, the OS was better in the low-risk group than in the high-risk group (*P* = 1.032e−04) (**Figure 6A**). Moreover, the AUCs were evaluated for 1-year (AUC = 0.724), 3-year (AUC = 0.716), and 5-year (AUC = 0.829) survival (**Figure 6B**). We next conducted univariate and multivariate Cox regression analyses to validate the predictive ability of the signature (**Figures 6D, E** **)**. The result reflected that the risk score plays an independent role in the prognosis of patients with CRC in the TCGA internal validation cohort. Then the total risk score was calculated based on the nomogram in the internal validation cohort. According to the median cutoff, we found that the low-risk group had a better OS than the high-risk group (*P* = 1.07e−05) (**Figure 6F**). Moreover, the AUCs evaluated for 1-, 3-, and 5-year OS were 0.837, 0.801, and 0.865, respectively (**Figures 6G–I**). The results of the calibration curve for OS prediction indicated the predicted value is consistent with the observed value (**Figures 6J–L**). The DCA curves of 1-, 3-, and 5-year OS predicted by the nomogram, the prognostic signature, and clinical variables are presented in **Supplementary Figures 3A–C**, which shows that the nomogram has a better net benefit at different threshold probabilities. The results indicate that the nomogram has good sensitivity and specificity.

**Figure 7:**
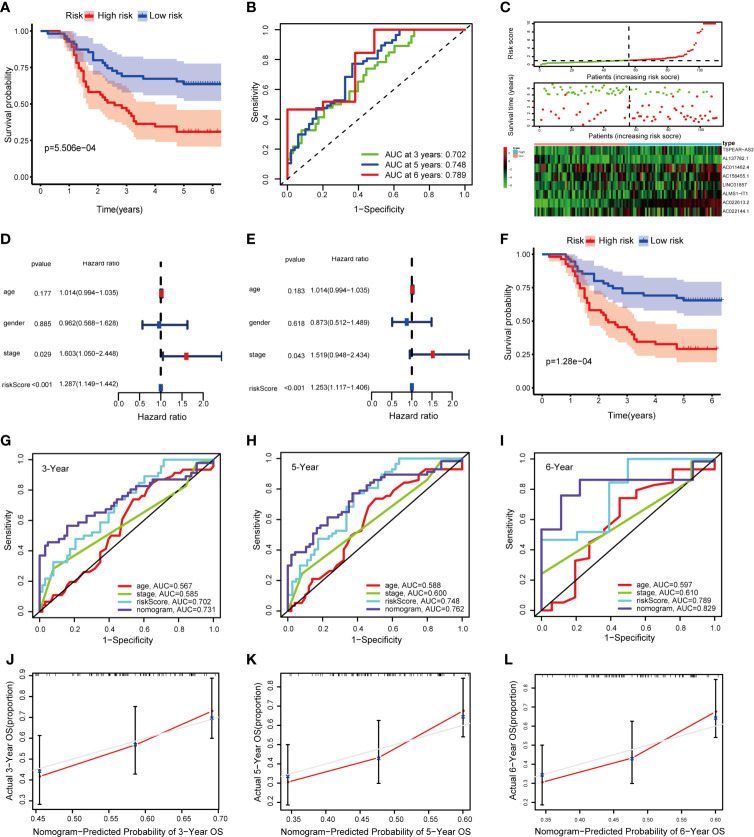
Validation of the signature and nomogram in the qRT-PCR external validation cohort. **(A)** Kaplan–Meier curves of patients with CRC based on the prognostic signature; **(B)** ROC curves of 1-, 3-, and 5-year OS predicted by the prognostic signature; **(C)** The distribution of the OS, risk score and lncRNA expression pattern; **(D, E)** Univariate and multivariate Cox regression analysis of OS-related variables; **(F)** Kaplan–Meier curves of patients with CRC based on the nomogram; **(G–I)** ROC curves of 1-, 3-, and 5-year OS predicted by the nomogram; **(J–L)** Calibration curves of 1-, 3-, and 5-year OS predicted by the nomogram.

To verify the result of bioinformatic analysis, we measured the expression level of the identified lncRNAs in 110 CRC samples by qRT-PCR. As expected, the OS was still better in the group with a low risk score than in the group with a high risk score (*P* = 5.506e−04) (**Figure 7A**). Moreover, the AUCs were evaluated for 3-year (AUC = 0.702), 5-year (AUC = 0.748), and 6-year (AUC = 0.789) survival (**Figure 7B**). And the distribution of the OS, risk score, and lncRNA expression pattern is shown in **Figure 7C**. The results of the univariate and multivariate Cox regression analyses showed the risk score works independently in the prognosis of patients with CRC (**Figures 7D, E** **)**. Similarly, the Kaplan–Meier survival analysis revealed that the OS was better in the group with a low total risk score than in the group with a high total risk score (*P* = 1.28e−04) (**Figure 7F**). Furthermore, the AUCs for 3-, 5-, and 6-year OS based on the nomogram were 0.731, 0.762, and 0.829, respectively (**Figures 7G–I**). Meanwhile, the calibration curve indicated great consistency between the predicted and actual observed values (**Figures 7J–L**). The DCA curves of 3-, 5-, and 6-year OS predicted by the nomogram, the prognostic signature, and clinical variables are presented in **Supplementary Figures 4A–C**, which shows that the nomogram has a better net benefit at different threshold probabilities. Taken together, these findings indicate that the nomogram shows excellent performance and reproducibility for OS prediction.

### Effects of lncRNA TSPEAR-AS2 on Fatty Acid Metabolism of Colorectal Cancer

**Figure 8:**
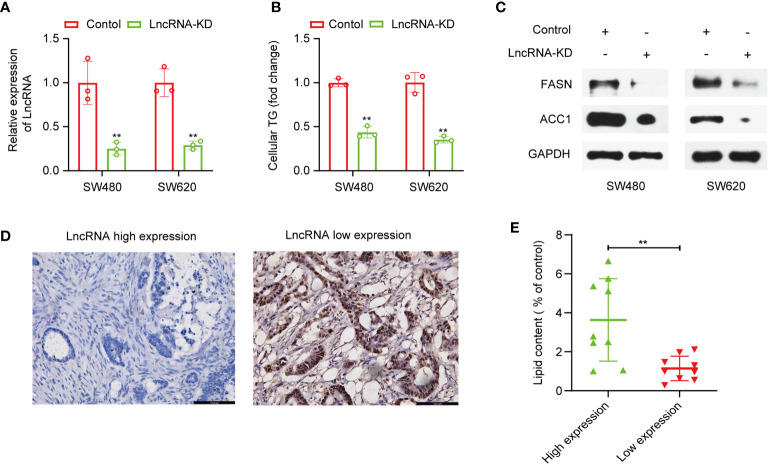
Effects of lncRNA TSPEAR-AS2 on fatty acid metabolism in CRC cells. **(A)** SW480 and SW460 cell lines were transfected with si-TSPEAR-AS2 (lncRNA-KD), and the knockdown efficiency was confirmed by qRT-PCR; **(B)** The effect of knockdown of lncRNA TSPEAR-AS2 on the triglyceride content in CRC cells; **(C)** FASN and ACC1 protein expression in CRC cells were examined by western blots. **(D)** Chromogenic *in situ* hybridization (CISH) was performed on clinical samples; **(E)** The lipid content (% of control) in lncRNA TSPEAR-AS2 high expression group and lncRNA TSPEAR-AS2 low expression group. **p < 0.01.

Furthermore, we attempted to validate the lncRNAs in the signature we constructed were fatty acid metabolism-related. Among the eight lncRNAs, lncRNA TSPEAR-AS2 has the highest expression difference between CRC samples and adjacent normal samples. So, we chose lncRNA TSPEAR-AS2 to validate the effects of lncRNA TSPEAR-AS2 on fatty acid metabolism in CRC cells. We transfected SW480 and SW620 cell lines with si-TSPEAR-AS2 (lncRNA-KD) and confirmed the knockdown efficiency by qRT-PCR **(** **Figure 8A** **)**. Afterward, intracellular triglyceride content was determined by the triglyceride assay kit. Interestingly, the result showed that knockdown of lncRNA TSPEAR-AS2 reduced the triglyceride content in CRC cells **(** **Figure 8B** **)**. Consistently, FASN and ACC1, the key enzymes of fatty acid synthesis metabolism, were significantly downregulated in lncRNA-KD CRC cells **(** **Figure 8C** **)**. Besides, to determine the high and low expression of lncRNA TSPEAR-AS2, we performed CISH on clinical samples (**Figure 8D**). Then we used Oil Red O staining to visualize the lipid deposits in tumor tissues with high and low expression of TSPEAR-AS2. The lipid content was determined by the absorbance of the extract at 540 nm. The result showed that the lipid content in lncRNA TSPEAR-AS2 high expression group was higher than that in lncRNA TSPEAR-AS2 low expression group (**Figure 8E**). Together, these findings suggest that lncRNA TSPEAR-AS2 plays an important role in the fatty acid metabolism of CRC.

## Discussion

CRC is a highly malignant tumor with high incidence and mortality (1). Therefore, the exploration of the underlying molecular mechanisms and the discovery of reliable prognostic biomarkers are urgent for improving prognosis evaluation and individualized treatment. Following in-depth studies of metabolic reprogramming, researchers gradually realized the importance of fatty acid metabolism in CRC (11, 13, 30, 31). However, our understanding of the association between lncRNA and fatty acid metabolism in CRC is limited. In this study, we constructed a fatty acid metabolism-related ceRNA network and identified eight fatty acid metabolism-related lncRNAs to build a prognostic signature for CRC. To achieve better predictive ability, we combined the prognostic signature with clinical variables to establish a nomogram with good reproducibility and reliability. Besides, we demonstrated the important function of lncRNA TSPEAR-AS2 included in the signature in the fatty acid metabolism of CRC. The current study suggests that a prognostic signature based on fatty acid metabolism-related lncRNAs can be utilized for prognosis stratification of patients with CRC. This prognostic signature will assist with elucidating the molecular mechanism of CRC and provide new ideas for fatty acid metabolism targeting therapies.

We first calculated the GSVA enrichment score of the fatty acid metabolism pathway of each patient with CRC. As a result, we found that the score was associated with the tumor stage and prognosis of patients by Kaplan–Meier survival analysis and correlation analysis. Furthermore, as crucial regulators of many physiological and pathological processes, lncRNAs also play irreplaceable roles in regulating the fatty acid metabolism of various cancers through ceRNA-related mechanisms (21, 22, 32). Shang Zhou et al. demonstrated that silencing PVT1 suppresses the expression level of FASN protein by sponging miR-195 in osteosarcoma (33). Moreover, in CRC, lncRNA SNHG16 may be used to target ceRNA up to 26 miRNA families to affect lipid metabolism-related genes up to 26 miRNA families to affect lipid metabolism-related genes (34). Next, we identified differentially expressed RNAs and establish a fatty acid metabolism-related ceRNA network. Though a few studies have constructed ceRNA networks in CRC (19, 25, 26), we are the first to establish a fatty acid metabolism-related ceRNA network in CRC. Here, we primarily focused on the lncRNAs in the ceRNA network. Surprisingly, in addition to the PVT1 and SNHG16 mentioned above, many of these lncRNAs have been explored to play important roles in fatty acid metabolism, such as NEAT1 (35), H19 (36), HOTAIR (37), HAGLR (38), and MALAT1 (39). This is sufficient to prove the credibility of the fatty acid metabolism-related ceRNA network we have built.

Finally, eight lncRNAs included in the ceRNA network were screened to construct a prognostic signature for CRC. Although several lncRNA-based prognostic signatures have been established in CRC, our study has outstanding strengths. First, the signature we constructed focuses on fatty acid metabolism-related lncRNAs, which contributes to therapy targeting fatty acid metabolism in CRC. Second, unlike previous studies using co-expression methods, we screened key DElncRNAs by establishing a fatty acid metabolism-related ceRNA network. Subsequently, we conducted a series of regression analyses to select OS-related lncRNAs, significantly improving the accuracy of the signature. Third, the signature has been verified in the TCGA internal validation cohort and the qRT-PCR external validation cohort, which ensures the clinical practicability of the signature. Among the eight lncRNAs, AL137782.1, AC022613.2, and AC022144.1 had not been previously reported. AC156455.1 has been shown to be an immune-related lncRNA by Pearson’s correlation test and was associated with prognosis in patients with colon adenocarcinoma (40). Robust evidence has shown that there is an intimate relationship between immunity and metabolism in cancer (41–43). We speculate that AC156455.1 may affect the immunity of CRC by regulating fatty acid metabolism, although further experiments are required to verify this speculation. Wei et al. indicated that AC011462.4 was associated with autophagy-related genes by Pearson’s correlation test (44). It is noteworthy that autophagy can regulate lipid acid metabolism (45), which is probably why AC011462.1 was involved in our fatty acid metabolism-related ceRNA network. Furthermore, ALMS1-IT1 was previously shown to be a prognostic signature in neck squamous cell carcinoma (46). LINC01857 is thought to promote breast cancer progression by regulating CREB1 activation *via* interacting with CREBBP (47). Additionally, it has also been suggested that LINC01857 may promote tumorigenesis and progression through ceRNA-related mechanisms in diffuse large B-cell lymphoma, glioma, and gastric cancer (48–50). Ma et al. indicated that high TSPEAR-AS2 expression was closely correlated with the prognosis of patients and promoted gastric cancer progression by regulating GJA1 and CLDN4 expression by sponging miRNA (51).

In addition, we revealed that after adjusting traditional clinical variables, the fatty acid metabolism-related lncRNAs prognostic signature remained work independently in the prognosis of patients with CRC; this indicates that the signature has the potential to improve the predictive ability of traditional clinical variables. Therefore, we constructed a nomogram that integrated the signature and clinical variables. Importantly, we verified the results in the TCGA internal validation cohort and the qRT-PCR external validation cohort of 110 CRC samples, which indicates good reproducibility and reliability of the prognostic model.

To validate the lncRNAs in the signature we constructed were fatty acid metabolism-related, we select lncRNA TSPEAR-AS2 to conduct functional experiments in CRC cells. The results showed that knockdown of lncRNA TSPEAR-AS2 decreased the triglyceride content in CRC cells. What’s more, the result of Oil Red O staining showed that the lipid content in lncRNA TSPEAR-AS2 high expression group was higher than that in lncRNA TSPEAR-AS2 low expression group. As we know, FASN and ACC1, the key enzymes of fatty acid synthesis metabolism, both control the lipogenesis, growth, and apoptosis of CRC cells (52, 53). Besides, the upregulation of FASN is related to metastasis in CRC (54, 55). Thus, we explored FASN and ACC1 expression in lncRNA-KD CRC cells and observed the downregulation of FASN and ACC1 expression when lncRNA TSPEAR-AS2 expression was downregulated. Taken together, these finding indicates that lncRNA TSPEAR-AS2 plays an important role in the fatty acid metabolism of CRC. Notably, in the ceRNA network we constructed, lncRNA TSPEAR-AS2 may control fatty acid metabolism by regulating long chain fatty acyl-CoA ligase 4 (ACSL4). ACSL4, as one member of Acyl-CoA synthetases (ACS) family, is responsible for the conversion of fatty acids to fatty acyl CoA esters (56). Present studies have demonstrated that the ACSL4 dysregulation is associated with the progression of many malignant tumors, including gastric cancer, prostate cancer, and colorectal cancer (57–59). What’s more, ACSL4 could regulate lipogenesis by affecting the expression of FASN and ACC1 (60, 61). Therefore, we speculated that lncRNATSPEAR-AS2 could affect fatty acid metabolism of CRC through the targeted regulation of ACSL4, which still needs further experiments to prove.

Our analysis has great importance in that it reveals a fatty acid metabolism-related ceRNA network and offers unique insights into the prognosis and treatment of CRC. However, there were several limitations in this study. First, the ceRNA-related mechanisms of fatty acid metabolism need to be clarified by further experiments *in vivo* and *in vitro*, and we are currently working on elucidating the mechanisms of the ceRNA network. Second, the information on CRC from the TCGA database is limited and incomplete, which may reduce the prediction accuracy of this nomogram, although it is hard to find an appropriate cohort in other databases to validate this nomogram. Third, though we validated the lncRNA signature and nomogram in the TCGA internal cohort and qRT-PCR external cohort, larger sample size CRC cohorts should also be used to provide further validation.

In summary, a fatty acid metabolism-related ceRNA network was constructed for the first time in the current study, which may elucidate the molecular regulatory mechanism of fatty acid metabolism in CRC. Moreover, this study contributes to our understanding of the regulation of fatty acid metabolism-related lncRNAs in CRC progression and provides novel potential biomarkers for prognosis and therapy.

## Data Availability Statement

The original contributions presented in the study are included in the article/**Supplementary Material**. Further inquiries can be directed to the corresponding authors.

## Ethics Statement

The studies involving human participants were reviewed and approved by Ethics Committee/Institutional Review Board of the Cancer Institute/Hospital, Peking Union Medical College and Chinese Academy of Medical Sciences (approval no. NCC2013RE-025). The patients/participants provided their written informed consent to participate in this study.

## Author Contributions

YP, CX, and YL designed and conducted the study. ZW and TZ carried out the validation. YP and TZ performed cellular experiments. YP, CX, and JW performed data analysis. YP and CX contributed equally to this work. YZ, MW, and YP wrote the manuscript. XL and KZ helped to improve the study. YP and TZ contributed to manuscript revision. All authors contributed to the article and approved the submitted version.

## Funding

This work was supported by grants from the Foundation of Science and Technology of Sichuan Province (2019YJ0635) and the National Natural Science Foundation of China (81502075).

## Conflict of Interest

The authors declare that the research was conducted in the absence of any commercial or financial relationships that could be construed as a potential conflict of interest.

## Publisher’s Note

All claims expressed in this article are solely those of the authors and do not necessarily represent those of their affiliated organizations, or those of the publisher, the editors and the reviewers. Any product that may be evaluated in this article, or claim that may be made by its manufacturer, is not guaranteed or endorsed by the publisher.

## Footnotes

1
https://cancergenome.nih.gov/

2
https://www.gencodegenes.org/

3
https://www.kegg.jp/

4
http://mirtarbase.mbc.nctu.edu.tw/

5
http://www.mirdb.org/

6
http://www.targetscan.org/

7
http://starbase.sysu.edu.cn/
