# Corrigendum: Fatty Acid Metabolism-Related lncRNAs Are Potential Biomarkers for Predicting the Overall Survival of Patients With Colorectal Cancer

**DOI:** 10.3389/fonc.2021.831864

**Published:** 2022-01-05

**Authors:** Yurui Peng, Chenxin Xu, Jun Wen, Yuanchuan Zhang, Meng Wang, Xiaoxiao Liu, Kang Zhao, Zheng Wang, Yanjun Liu, Tongtong Zhang

**Affiliations:** ^1^ The Center of Gastrointestinal and Minimally Invasive Surgery, Department of General Surgery, The Third People’s Hospital of Chengdu, The Affiliated Hospital of Southwest Jiaotong University, Chengdu, China; ^2^ Department of Colorectal Surgery, National Cancer Center, National Clinical Research Center for Cancer, Cancer Hospital, Chinese Academy of Medical Sciences and Peking Union Medical College, Beijing, China; ^3^ Medical Research Center, The Affiliated Hospital of Southwest Jiaotong University, The Third People’s Hospital of Chengdu, Chengdu, China

**Keywords:** colorectal cancer, fatty acid metabolism, long noncoding RNA, ceRNA network, signature, nomogram

In the published article, there was an error in affiliation 1. Instead of “The Center of Gastrointestinal and Minimally Invasive Surgery, The Affiliated Hospital of Southwest Jiaotong University, The Third People’s Hospital of Chengdu, Chengdu, China”, it should be “The Center of Gastrointestinal and Minimally Invasive Surgery, Department of General Surgery, The Third People’s Hospital of Chengdu, The Affiliated Hospital of Southwest Jiaotong University, Chengdu, China”.

The authors apologize for this error and state that this does not change the scientific conclusions of the article in any way. The original article has been updated.

## Publisher’s Note

All claims expressed in this article are solely those of the authors and do not necessarily represent those of their affiliated organizations, or those of the publisher, the editors and the reviewers. Any product that may be evaluated in this article, or claim that may be made by its manufacturer, is not guaranteed or endorsed by the publisher.

