# Supplementary material for: Fatty Acid Metabolism-Related lncRNAs Are Potential Biomarkers for Predicting the Overall Survival of Patients With Colorectal Cancer
Source: Front Oncol. 2021 Aug 11;11:704038. doi: 10.3389/fonc.2021.704038 (PMC8386021; doi:10.3389/fonc.2021.704038)
Supplement: Supplementary file 1 [file DataSheet_1.docx]

Supplementary Material

# Supplementary Tables

| **Supplementary Table 1. Primers used in the study.** | |  |
| --- | --- | --- |
| Gene | Primer sequences |  |
|  | Forward | Reverse |
| AC156455.1 | CAGCTGTGGCACCAAAAGTC | TGCTTGGCCAGAGTTAGGTG |
| AC011462.4 | ATGACGACCCTGTTGGCATT | CGTGGGTCATGTGAGTGTGA |
| TSPEAR-AS2 | TCATCTTTGGGTGGAGCCAC | TGACCCAGTCTCCTGTCAGT |
| AL137782.1 | CAACTCACGTCAGCCTCAGT | AGAACAACTGCTCCAGAGGC |
| LINC01857 | TTAAGAAGCTCCACTGCGCT | ACATCACGCAGAGGCTTTGA |
| ALMS1-IT1 | CATGCACACAGTTGCTGAGG | TACCCGTCAAGAACCACTGC |
| AC022613.2 | GCACTGCACAGGAAACACAG | GAAGCAGGGAAGTGTGAGCT |
| AC022144.1 | TAATTTGGCGCTCCCCACTT | GATGCCCTTCCAGTTCCCAA |
| GAPDH | ACCACAGTCCATGCCATCAC | TCCACCACCCTGTTGCTGTA |

# Supplementary Figures


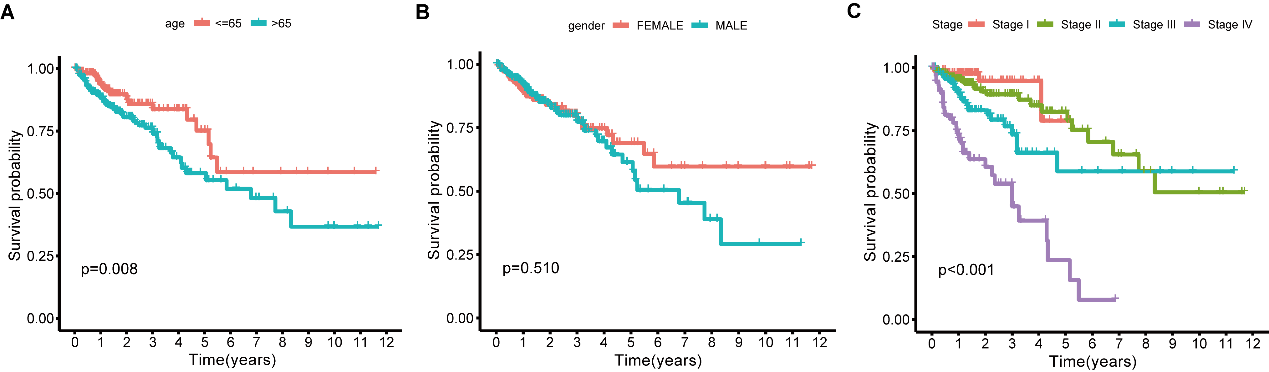


**Supplementary Figure 1.** Identification of OS-related clinical variables by Kaplan-Meier analysis. (A) Kaplan-Meier curves stratified by age; (B) Kaplan-Meier curves stratified by gender; (C) Kaplan-Meier curves stratified by tumor stages;


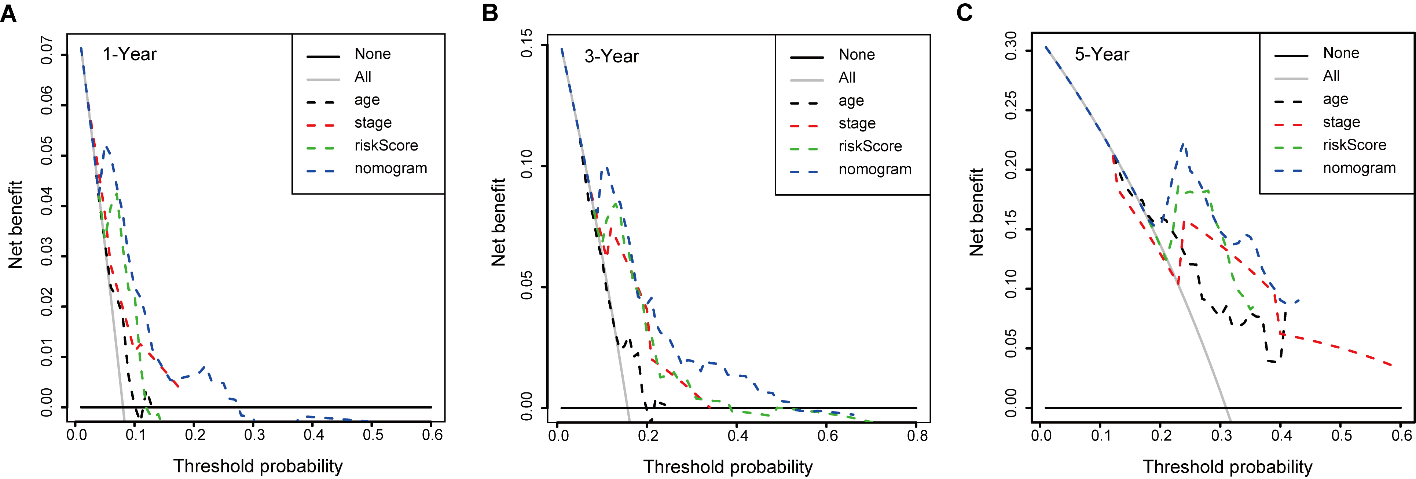


**Supplementary Figure 2.** DCA curves. **(A–C)** DCA curves of 1-, 3-, and 5-year OS predicted by the nomogram, the prognostic signature, and clinical variables in the training cohort.


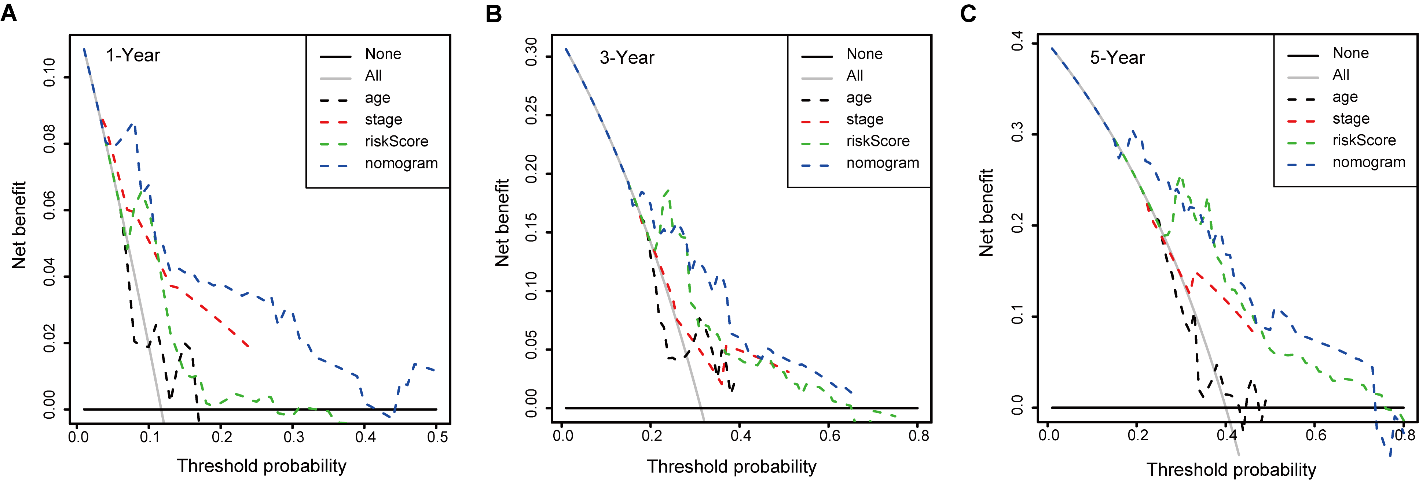


**Supplementary Figure 3.** DCA curves. **(A–C)** DCA curves of 1-, 3-, and 5-year OS predicted by the nomogram, the prognostic signature, and clinical variables in the TCGA internal validation cohort.


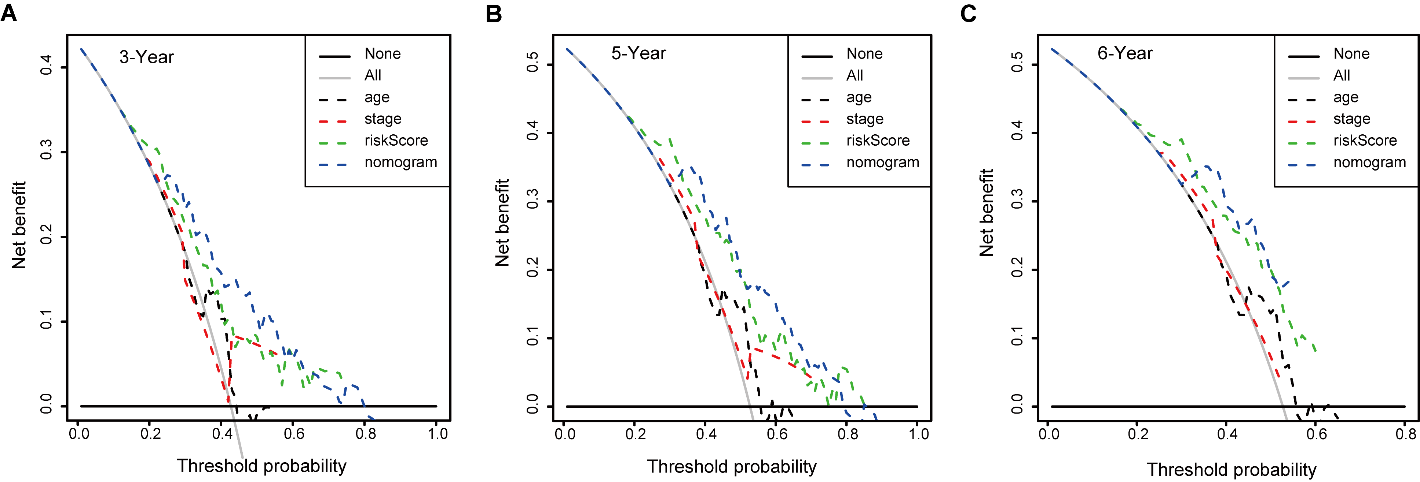


**Supplementary Figure 4.** DCA curves. **(A–C)** DCA curves of 3-, 5-, and 6-year OS predicted by the nomogram, the prognostic signature, and clinical variables in the qRT-PCR external validation cohort.
